# RAFT Hydroxylated Polymers as Templates and Ligands for the Synthesis of Fluorescent ZnO Quantum Dots

**DOI:** 10.3390/nano12193441

**Published:** 2022-10-01

**Authors:** Leire San José, Olga García, Isabel Quijada-Garrido, Mar López-González

**Affiliations:** Group of Nanohybrids and Interactive Polymers, Instituto de Ciencia y Tecnología de Polímeros (ICTP-CSIC), C/Juan de la Cierva, 3, 28006 Madrid, Spain

**Keywords:** ZnO QDs, hydroxylated RAFT polymers, nanohybrids, fluorescent nanomaterials

## Abstract

The remarkable photoluminescent properties, biocompatibility, biodegradability, and antibacterial properties of zinc oxide quantum dots (ZnO QDs) coupled with their low cost and nanoscale size guarantee bio-related and technological applications. However, the effect of the polymeric ligand during synthesis has hardly been investigated compared to other less environmentally friendly QDs. Thus, the objective of this work was to focus on the synthesis of fluorescent hybrid ZnO QDs by the sol-gel method using different polymers with hydroxyl groups as templates and ligands to obtain stable particles in different media. For this purpose, well-defined hydroxylated statistical polymers and block copolymers were synthesized using reversible-addition fragmentation chain transfer (RAFT) polymerization to establish the influence of molecular weight, hydrophobic/hydrophilic balance, and polymer architecture on the colloidal and photophysical properties of the synthesized hybrid ZnO QDs. Dynamic light scattering (DLS), TEM, and X-ray diffraction measurements indicated the formation of stable nanoparticles of a few nanometers. A remarkable enhancement in terms of fluorescence was observed when ZnO QDs were synthesized in the presence of the hydroxylated homopolymers and even more so with block copolymers architecture. Organosilanes combined with the hydroxylated polymers were used to improve the colloidal stability of ZnO QDs in aqueous media. These samples exhibited uniform and stable enhanced photoluminescence for nearly five months of being investigated. Among other applications, the hybrid ZnO QDs synthesized in this work exhibit high selectivity to detect Cr^6+^, Fe^2+^, or Cu^2+^ in water.

## 1. Introduction

Research on zinc oxide quantum dots (ZnO QDs) has received considerable interest due to their photoluminescent (PL) properties and nanoscale size [1]. Being an inexpensive and environmentally friendly material makes it an attractive option for its applications in the biological field for the delivery of anti-tumor drugs [2,3,4], as an antimicrobial agent [5,6,7], or in bioimaging [8], but also in other applications such as anti-counterfeiting technology [9], gas sensors [10,11], the detection of metal ions [12,13,14,15,16,17,18], photocatalysis [17,18,19], and optoelectronics [20,21,22,23,24,25]. A recent review on the synthesis, properties, and applications of ZnO QDs was carried out by Singh et al. [1].

The sol-gel method is the most extended method for the synthesis of ZnO QDs. Typically, this process involves the hydrolysis of zinc acetate in ethanol under sonication, generating nanoparticles between 3–5 nm with an emission about 500–550 nm [26]. However, the as-synthesized particles do not have colloidal stability, and after a short period of time the particles undergo the process known as Ostwald ripening and end up precipitating in the reaction medium [27]. To overcome this problem, some strategies have been applied to highlight the synthesis of ZnO QDs using triethyleneglycol or tetraethyleneglycol as reaction media [28,29] and through the use of silanes [25] and polymer ligands of different nature [12,27,30]. Furthermore, to provide colloidal stability in the reaction medium, polymers can confer other properties, such as solubility in different media, an increase in terms of biocompatibility, and functionalization capacity, among others [31]. Thus, Laopa and Vilaivan [12] found that ZnO QDs can be stabilized by a cationic copolymer via an ionic interaction with the citrate ligand, enhancing the fluorescence quantum yield (Φ_F_) up to 27–32%. Zheng et al. [30] investigated the role of double hydrophilic block copolymers, consisting of a polyethylene glycol (PEG) stabilizing block and a second block bearing chemical groups with affinity for the ZnO surface (either carboxylic or phoshonic acid), on the stabilization and luminescence of ZnO QDs in THF and water. The hydrolysis of zinc methacrylate and in situ polymerization of the methacrylic surface ligand with a polyethylene glycol methyl ether methacrylate produced ZnO@polymer core-shell nanoparticles with tunable PL [3] and a high Φ_F_. In addition to these functionalities (carboxylic, phosphonic groups, etc.), the hydroxyl groups of poly(vinyl alcohol) PVA have shown avidity for the surface of ZnO QDs through the formation of hydrogen bonds, giving rise to very stable nanocomposites [32]. This leads us to hypothesize that other polymers with hydroxyl groups could be suitable ligands for the stabilization of ZnO QDs. Controlled living radical polymerization (CLRP), such as RAFT, allows for a wide variety of monomers with hydroxyl groups, which opens up a range of polymers with control of their chemical composition, molecular weight, and architecture. Furthermore, RAFT polymerization is ideal for the synthesis of functional polymers to hybridize inorganic nanostructures [33,34]. With this background, in this work, we focused on the synthesis of fluorescent hybrid ZnO QDs in the presence of polymers with multihydroxyl groups as templates and ligands in order to obtain stable particles in the nanoscale. Well-defined multihydroxylated statistical homopolymers and block copolymers, including a hydrophilic poly(polyethylene glycol methacrylate) block and a second more hydrophobic block comprising monomers with hydroxyl groups able to interact with the ZnO surface, were synthesized by RAFT polymerization. The influence of the molecular weight, polymer architecture, and hydrophobic/hydrophilic balance on the colloidal and photophysical properties of the ZnO QDs was investigated in detail. However, while polymers can improve colloidal stability in the reaction medium and enhance the Φ_F_, achieving stability in aqueous media for long periods of time remains a significant challenge, since the addition of small amounts of water to the medium can cause the precipitation of ZnO with the unavoidable loss of luminescence. So, the most successful and robust method to overcome this drawback is the silanization of the ZnO QDs’ surfaces by means of alkoxysilanes [35,36,37]. The dense cross-linked network around the ZnO nanoparticle will prevent its evolution and loss of luminescence over time. Since silanes can react with hydroxyl groups during the condensation, in this contribution, we explored the combination of hydrophobic and hydrophilic silanes with hydroxylated polymers to create a more versatile and protective coating. The hydroxyl groups of the copolymers can covalently attach to the siloxane network, which provides a polymeric shell to the silanized ZnO QDs, which in turn improves the colloidal stability, providing a long-lasting fluorescence in aqueous medium. Due to the robustness of these nanoparticles, among other promising applications in the fields of biotechnology and optoelectronics, in this work, we took advantage of the fluorescence quenching experienced by QDs in the presence of metals for the purpose of detecting pollutants. Unfortunately, the contamination of water with different metals from industry is currently a significant concern, so the development of robust and economical detection methods is critical from an environmental point of view.

## 2. Materials and Methods

### 2.1. Materials

Poly(propylene glycol) methyl ether methacrylate (PPGMA), average M_n_~375 (Sigma-Aldrich, Darmstadt, Germany), hydroxypropyl methacrylate (HPMA) (Sigma-Aldrich, 97%), poly(ethylene glycol) methyl ether methacrylate (PEGMEMA), average M_n_~500 (Sigma-Aldrich), (4-cyano-4-[(dodecylsulfanylthiocarbonyl) sulfanyl]pentanoic acid (CDTPA) (Sigma-Aldrich, 97%), 4,4′-azobis(4-cyanovaleric acid) (ACVA) (Sigma-Aldrich, ≥98%) for polymers synthesis. Zinc acetate dihydrate Zn(CH_3_COO)_2_·2H_2_O (Sigma-Aldrich, ≥99%), potassium hydroxide (KOH) (Panreac, Barcelona, Spain, 85%), N-(trimethoxysilylpropyl)ethylenediamine triacetic acid trisodium salt (TMSPEDATA) (Gelest, Morrisville-PA, USA 35% in water), trimethoxy(octadecyl)silane (TMODS) (Sigma-Aldrich), (3-aminopropyl)triethoxysilane (APTES) (Sigma-Aldrich, ≥ 99%) for ZnO QDs synthesis. Rhodamine 6G for Φ_F_ determination was provided by Luxottica-Exciton (Lockbourne-OH, USA). Metallic salts for quenching studies: lithium bromide (LiBr, Honeywell, ≥99%), magnesium oxide (MgO, Sigma-Aldrich, >97%), potassium carbonate (K_2_CO_3_, Panreac, ≥99.9%), calcium hydride (CaH_2_, Sigma-Aldrich, >95%), potassium chromate (K_2_CrO_4_ Sigma-Aldrich, >99%), manganese(II) sulfate monohydrate, (MnSO_4_·H_2_O, Sigma-Aldrich, >99%), iron(II) chloride tetrahydrate (FeCl_2_·4H_2_O, Aldrich, ≥99%) and iron(III) chloride hexahydrate (FeCl_3_·6H_2_O, Aldrich, ≥99.9%), cobalt nitrate hexahydrate (Co(NO_3_)_2_·6H_2_O, Panreac, ≥99.9%), nickel nitrate hexahydrate (Ni(NO_3_)_2_·6H_2_O, Fluka, Buchs, Switzerland, ≥98.5%), copper(II) perchlorate hexahydrate (Cu(ClO_4_)_2_·6H_2_O, Aldrich, ≥98%), mercury(II) chloride (HgCl_2_, Panreac, ≥99.9%) and lead(II)nitrate (Pb(NO_3_)_2_, Aldrich, ≥99%), were all used as received. Absolute ethanol (Merck, Darmstadt, Germany, 99.8%), methanol (Merck, ≥99.9%), Milli-Q water, hexane (Scharlau, Barcelona, Spain, HPLC grade). All reagents were used as received without further purification.

### 2.2. Synthesis of Homopolymers via RAFT Polymerization

pPPGMA, pHPMA, and pPEGMEMA homopolymers, from now on denoted as PG, HP, and EG, respectively, were synthesized by RAFT polymerization, for further use, namely both hydrophobic PG and HP as a coating for ZnO nanoparticles, due to the hydroxyl group they contain, and hydrophilic EG for further block copolymers synthesis.

**Scheme 1:**
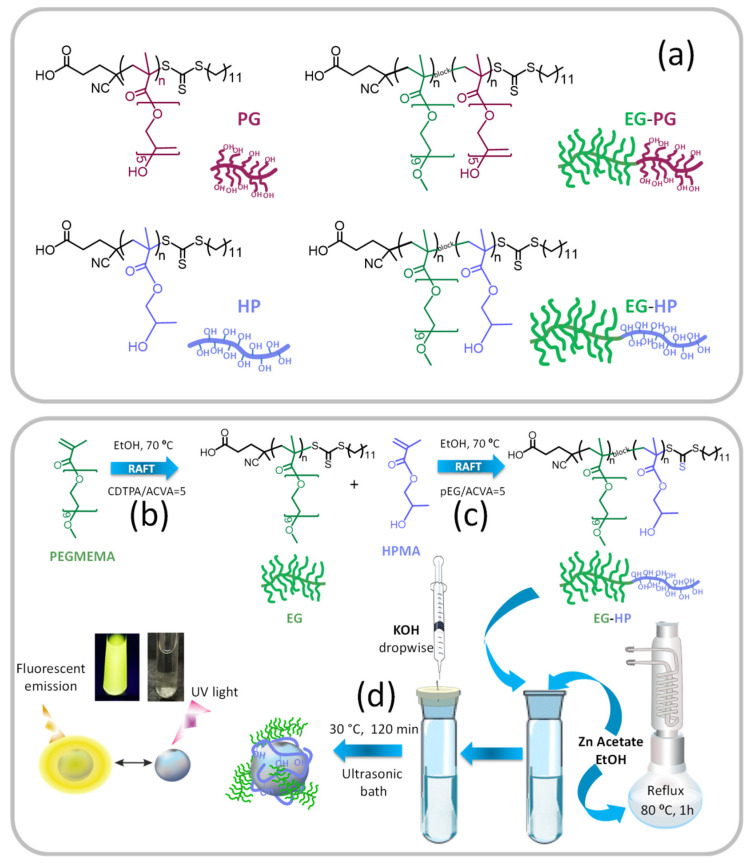
(**a**) RAFT hydroxylated homopolymers and block copolymers used as template for the ZnO QDs synthesis; (**b**) procedure for poly(polyethylene glycol methacrylate) (EG) synthesis; (**c**) synthesis of poly(polyethylene glycol methacrylate-*b*-poly(hydroxypropyl methacylate) (EG-HP) block copolymer; and (**d**) synthesis of luminescent hybrid ZnO QDs protected with EG-HP block copolymer.

PG, HP, and EG homopolymers (Table 1) of different molecular weights were synthesized in ethanol at 70 °C by setting different monomer/CDTPA RAFT ratios (from 25 to 75) and reaction times (2, 3, 4, 5 h). Their chemical structures are shown in Scheme 1a,b. A typical protocol for the synthesis of sample PG_14k_ (subscript indicates theoretical M_n_ in kg mol^−1^) is shown below: CDTPA RAFT agent (0.0333 g, 0.08 mmol) was added to PPGMA (1.546 g, 4 mmol) and dissolved in absolute ethanol (2.3 g, 48.8 mmol) in a 20 mL glass tube, followed by the addition of ACVA initiator (5.98 mg, 0.016 mmol, CDTPA/ACVA ratio = 5). This resulted in a yellow solution that was purged with nitrogen for 20 min in an iced-water bath followed by another 10 min at room temperature. The sealed tube was immersed in an oil bath at 70 °C and magnetically stirred for 5 h. After that, the reaction was stopped by exposure to air and immersion in an iced-water bath. The product was diluted in methanol and precipitated for three times in hexane in order to remove the unreacted monomers. Purified polymers were high vacuum dried and stored at 4 °C. The estimated monomer conversion according to ^1^H-NMR spectra is gathered in Table 1.

### 2.3. Synthesis of Copolymers via RAFT Polymerization

A series of pPEGMEMA-*b*-pPPGMA (EG-PG) and pPEGMEMA-*b*-pHPMA (EG-HP) copolymers of several molecular weights (Table 1) were synthesized to be used as ZnO nanoparticles templates and ligands on further reactions. Their structures are shown in Scheme 1a.

The two series of copolymers were synthesized using the three EG homopolymers collected in Table 1. The EG macro-CTA/monomer (PPGMA or HPMA) ratio was fixed to 50 or 75 in a reaction of 3 h at 70 °C. The protocol for the synthesis of the copolymers is presented in Scheme 1c and is shown below with the sample EG_21k_-PG_14k_. Thus, EG_21k_ (0.618 g, 0.02 mmol) and PPGMA (0.394 g, 1.02 mmol) were dissolved in absolute ethanol (1.5 g, 32.6 mmol) in a 20 mL glass tube, followed by the addition of ACVA initiator (1.17 mg, 0.0041 mmol, EG_21k_/ACVA ratio = 5). This resulted in a yellow solution that was purged with N_2_ gas for 20 min and placed in an iced-water bath followed by another 10 min at room temperature. The sealed tube was immersed in an oil bath at 70 °C and magnetically stirred for 3 h. After that, the reaction was exposed to air and immersed in an iced-water bath. An aliquot of the reaction crude was taken to determine the monomer conversion by ^1^H-NMR. The reaction crude was diluted in methanol and precipitated in hexane, this process was repeated for three times, in order to remove the unreacted monomers. The precipitate was high vacuum dried and stored at 4 °C.

### 2.4. Synthesis of Fluorescent ZnO QDs

The synthetic procedure for the ZnO QDs synthesis [26], based on the sol-gel method, is presented in Scheme 1d. The procedure begins with the preparation of the organometallic precursor, i.e., 25 mL of 0.06 M solution of zinc acetate dihydrate (Zn(CH_3_COO)_2_·2H_2_O) in absolute ethanol. The solution, in a round bottom flask, is refluxed at 80 °C and magnetically stirred for 1 h. After cooling to room temperature, 5 mL of the solution are taken and transferred to a glass reaction tube, followed by the addition of the polymer, silane, or silane/polymer chosen for each experiment. The tube is then sealed and placed inside an ultrasonic bath at 30 °C, where KOH 0.9 M solution is added dropwise in an OH^-^/Zn^2+^ molar ratio of 2:1. The obtained clear solution with the coated ZnO QDs is cooled down in an iced-water bath, stored at 4 °C, and protected from light. For XRD and FTIR analysis, samples were concentrated in ethanol and precipitated in hexane to remove the excess precursors.

### 2.5. Characterization and Properties

^1^H-NMR spectra were recorded in DMSO-d6 and CDCl_3_ (depending on polymer solubility) solvents using a Bruker Avance III-HD-400 spectrometer. Molecular weight distributions and dispersity (Ð = M_w_/M_n_) of homopolymers and copolymers were determined by gel permeation chromatography (SEC) on a Perkin Elmer series 200 system equipped with a refractive index detector and heated columns at 70 °C. DMF stabilized with 0.1 wt% of LiBr was used as the mobile phase at 0.8 mL min^−1^ and 70 °C using poly(methyl methacrylate) (PMMA) standards (Polymers Laboratories LTD, Shropshire, United Kingdom) for the calibration. Infrared measurements were carried out by Perkin-Elmer Spectrum Two FTIR spectrometer fitted with an attenuated total reflectance (ATR) accessory. The crystalline structure of the ZnO QDs was analyzed by X-Ray diffraction (XRD). Diffractograms were recorded in the reflection mode by using a Bruker D8 Advance diffractometer provided with a PSD Vantec detector (from Bruker, Madison, WI). CuKα radiation (λ = 0.1542 nm) was used, operating at 40 kV and 40 mA. The equipment was calibrated with different standards. The diffraction scans were collected within the range of 2θ = 4−80°, with a 2θ step of 0.024° and 0.5 s per step. ZnO QDs morphology and size were determined by transmission electron microscopy (TEM) in a JEOL JEM-2100 HT microscope operated at 200 kV and equipped with a LaB6 gun, an CCD ORIUS SC1000 (Model 882) camera, a STEM unit with an ADF detector, and a point resolution of 0.25 nm. This microscope is located at ICTS Centro Nacional de Microscopía Electrónica at UCM (Madrid, Spain). Mean particle size was obtained by measuring at least 120 particles. The hydrodynamic size of the nanoparticles was determined by dynamic light scattering (DLS)**.** Diluted samples in ethanol were measured at 20 °C to determine the hydrodynamic size as the number distribution by a Zetasizer Nano ZS instrument (Malvern Instruments Ltd., Malvern, UK). Malvern Dispersion Software was used for data acquisition and analysis.

The emission spectra of hybrid ZnO QDs were recorded on a Perkin Elmer FL6500 spectrophotometer in ethanol at the excitation wavelength of 365 nm, using a Rhodamine 6G solution in ethanol as standard. The absorption spectra of the synthesized ZnO QDs and Rhodamine 6G in ethanol were recorded on a UV/Vis NanoDrop One Thermo-Scientific spectrometer. Fluorescence quantum yield (Φ_F_) of the ZnO QDs was calculated by the relative method using the following equation applicable for diluted solutions (absorbance ≤ 0.10, at the excitation wavelength):

(1) $$\Phi_{F}=\frac{I_{A\left(s\right)\;}I_{E}{\;\eta }^{2}}{I_{A\;}I_{E\left(s\right)\;}\eta_{\left(s\right)}^{2}}\Phi_{s}$$

where Φ_S_ is the quantum yield of the Rhodamine 6G standard in ethanol (Φ_S_ = 95%) [38], $I_{A\;}$refers to the absorbed light intensity at lambda excitation (365 nm) by the sample, and $I_{A\;\left(s\right)}$ is the same by the Rhodamine 6G, calculated from:

(2) $$I_{A}=1-10^{-\mathrm{Abs}}$$

$I_{E}$ and $I_{E\left(s\right)}\;$are the integrated emitted fluorescence intensity of the sample and the Rhodamine 6G, respectively, η is the refraction index of the sample solution, and η_(s)_ the refraction index of the Rhodamine 6G ethanolic solution.

Experiments to investigate the use of hybrid ZnO QDs as sensors for metal detection were carried out by incubating 50 μL of the sample reaction diluted 1:10 in water for 1 h, with 50 μL of different salts solution to obtain a final concentration between 5 and 100 μM, and studying the resulting fluorescence emission signal.

## 3. Results and Discussion

### 3.1. Synthesis of RAFT Hydroxylated Copolymers

Since previous reports indicated that polymers with hydroxyl groups as PVA are useful for the stabilization of ZnO QDs, in the present work, we propose the synthesis of hydrophobic and amphiphilic polymers multi-functionalized with hydroxyl groups to act as templates in the synthesis of hybrid ZnO QDs.

To this end, hydrophobic methacrylic homopolymers (PG and HP) and amphiphilic block copolymers with either a polypropylene glycol side chain (EG-PG) or hydroxypropyl (EG-HP) side chain were synthesized (Scheme 1a) by RAFT polymerization. For the homopolymers and EG first block synthesis, CDTPA was used as a RAFT agent and a CDTPA/ACVA, at ratio of 5 in ethanol at 70 °C, was also used. Ethanol was selected as a solvent since the synthesis of the ZnO QDs was also to be carried out in this alcohol, so traces of residual solvent would not affect this reaction. In Table 1, the polymer composition, conversion, and molecular weight parameters are collected and in Figure S1 ^1^H-NMR spectra corresponding to representative EG, HP, and PG homopolymers are shown. As it can be seen in Table 1, the hydroxylated homopolymers synthesized by RAFT present low dispersity (Ð), indicating that polymerization was well controlled, despite PG homopolymers exhibiting a higher Ð than HP and EG homopolymers. Block copolymers were synthesized from the extension of EG of different molecular weights, allowing for the obtaining of the copolymers collected in Table 1, which also exhibit a low Ð (lower than 1.3), indicating the suitability of the polymerization conditions. Figure S2 displays selected SEC chromatograms for representative EG-HP and EG-PG block copolymers, while ^1^H-NMR spectra of EG macro-RAFTs and block copolymers, displaying their characteristic chemical shift, are presented in Figures S1 and S3.

### 3.2. ZnO QDs with Hydroxylated Polymers as Ligands

The synthesis was carried out using the classical sol-gel method since it has the lowest cost and is a simple, repetitive, and reproducible synthesis [20]. A solution of zinc acetate in ethanol was hydrolyzed by adding KOH in the absence or in the presence of different polymers with hydroxyl groups, while keeping the solution in an ultrasonic bath (frequency of 37 kHz). The process is streamlined in Scheme 1d and sample properties are displayed in Table 2. The reaction was carried out at 30 °C since preliminary experiments (Figure S4) indicated that increasing synthesis temperature leads to a decrease of PL intensity, a red shift of the fluorescence maximum, and an increase in the particle size, in agreement with previous studies [39].

**Figure 1:**
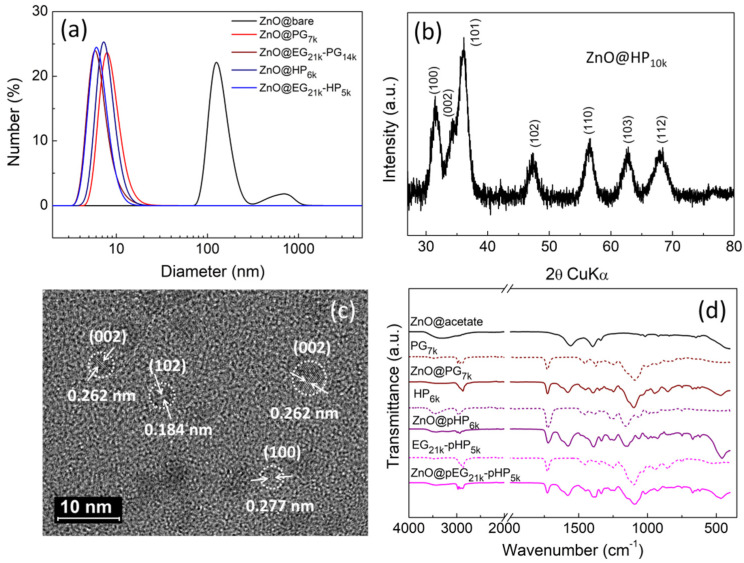
(**a**) Size by number distribution (%) from DLS measurements of the bare ZnO QDs sample and representative hybrid ZnO QDs coated with hydroxylated homopolymers and block copolymers; (**b**) X-ray diffraction patterns corresponding to representative hybrid ZnO QDs showing the wurtzite crystalline structure; (**c**) TEM image corresponding to ZnO QDs synthesized in the presence of HP_7k_ polymeric coatings; and (**d**) ATR-FTIR spectra corresponding to bare ZnO QDs and representative ZnO QDs coated with hydroxylated homopolymers and block copolymers.

The formation of ZnO QDs with hydrodynamic diameters under 10 nm in the presence of RAFT hydroxylated polymer ligands is clearly observed in the DLS measurements shown in Figure 1a for a number of representative hybrid ZnO QDs in ethanol, while the dispersion of uncoated ZnO QDs (ZnO@bare) in ethanol results in large aggregates.

In Figure 1b, the XRD spectrum shows the typical pattern for ZnO nanoparticles, with the broadening of the peaks due to the nanometric size [40]. All of the diffraction peaks were consistent with the database on JCPDS file No. 80-0075 and could be indexed according to a wurtzite structure, although the phase of Zn(OH)_2_ could not be completely ruled out. According to the Debye−Scherrer equation, a mean size of 5.04 nm was obtained for ZnO@HP_10k_ nanoparticles in Figure 1b. This value was calculated from the diffraction peaks at 2θ = 47.4 and 56.6° assignable to the (102) and (110) planes, respectively [41]. Figure 1c displays TEM images corresponding to ZnO@HP_7k_ nanoparticles, revealing the formation of spherical nanoparticles that are on average 4.5 nm size, in good agreement with the XRD. Most of the d-spacing of the (002) (0.26 nm) and (100) (0.28 nm) planes, consistent with wurtzite, are indicated by parallel lines in Figure 1c. The presence of homopolymer and copolymer ligands on the ZnO QDs surfaces was ascertained by the analysis of the FTIR spectra shown in Figure 1d. An FTIR spectrum of bare ZnO synthesized without polymer exhibits bands at 1560 and 1400 cm^−1^, corresponding to the stretching vibration –COO^-^ of residual potassium acetate, a broad band from 3600 to 2700 cm^−1^, assigned to the hydroxyl groups of ethanol still adsorbed on the ZnO surface, as well as a broad band around 400 cm^−1^, attributed to the Zn-O bonds [42]. The FTIR spectra corresponding to ZnO QDs with polymer coating exhibit vibration bands corresponding to the polymeric ligands; notably, at 3000 to 2800 cm^−1^, the C-H stretching vibrations correspond to the CH_3_, CH_2_, and CH of the polymer backbone and isopropyl groups of the side chain, and at 1725 cm^−1^, they correspond to the strong stretching vibration of -C=O group. At approximately 1090 cm^−1^, the typical C-O-C stretching of ether appears in ZnO QDs functionalized with polypropylene glycol and polyethylene glycol polymers. In addition, bands at 1560 and 1400 cm^−1^ corresponding to residual potassium acetate still appear. The band attributed to the Zn-O appears at around 460–467 cm^−1^ in ZnO nanoparticles with polymer ligands.

**Figure 2:**
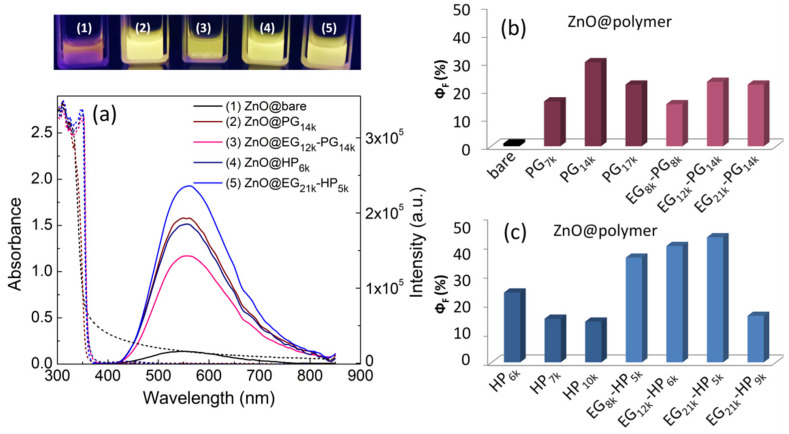
(**a**) Absorbance (dashed lines) and emission fluorescence (λ_exc._ = 365 nm) (solid lines) bands from ethanol solutions of ZnO QDs with/without coating as specified in the legend, after 24 h of their synthesis; the insert at the top shows photos of these ZnO QDs samples under UV light (365 nm). (**b**) Φ_F_ (%) for ZnO QDs synthesized without polymer and in the presence of PG_x_ homopolymers and EG_y_-PG_x_ block copolymers of different molecular weights. (**c**) Φ_F_ (%) for ZnO QDs synthesized in the presence of HP_x_ homopolymers and EG_y_-HP_x_ block copolymers of different molecular weights.

In Figure 2a, the absorbance spectrum corresponding to ZnO QDs without a polymer ligand (ZnO@bare) is compared with spectra of the particles synthesized in the presence of representative PG, EG-PG, HP, and EG-HP copolymers (dashed lines). ZnO QDs with polymer protection exhibit a sharp increasing of absorbance below 360 nm. The absence of absorbance above 360 nm for the samples synthesized in the presence of polymers indicates that there are not aggregates, in opposite to the sample synthesized without any ligand. Emission curves for a series of hybrid ZnO QDs with different polymer coatings are presented in Figure 2a as solid lines and compared with ZnO QDs without coating. For all the synthesized hybrid ZnO QDs the emission is centered between 549–564 nm after excitation at 365 nm, regardless of the presence or nature of the polymer employed in the synthesis. However, the photoluminescence emission experiences a strong enhancement with the addition of the different homopolymers and copolymers to the reaction batch (Table 2). To quantify this effect, the Φ_F_ (%) values obtained are shown in the last column of Table 2 and represented, for clarity, in the form of a bar chart in Figure 2b, for ZnO QDs coated with PG_x_ homopolymers and EG_y_-PG_x_ block copolymers, and in Figure 2c, for ZnO QDs coated with HP_x_ and EG_y_-HP_x_ block copolymers, where *x* and *y* represent the molecular weight of each block.

As it can be seen in Figure 2 and Table 2, the quantum yield (Φ_F_) of ZnO@polymer noticeably increases with the polymer protection, in agreement with previous reports [12,27]. Comparing the results obtained with the different polymeric coatings, polypropylene glycol methacrylate homopolymers (PG_x_) provide comparable protection for ZnO QDs to that provided by hydroxypropyl methacrylate homopolymers (HP_x_), which results in similar quantum yields (Φ_F_). However, the Φ_F_ does not improve when ZnO nanoparticles are hybridized with EG-PG block copolymers. On the other hand, EG-HP block copolymers clearly increase the Φ_F_ over the HP homopolymers; in special ZnO QDs coated with EG_21k_-HP_5k_ block copolymer, the Φ_F_ reached 43%. In the case of HP polymers, a clear influence in terms of the molecular weight is observed, specifically, the increase in the size of the hydrophilic EG block improves the fluorescence quantum yield. On the contrary, the increase in the size of the more hydrophobic hydroxylated HP block produces a detriment in the quantum yield for both homo- and block copolymer ligands (Table 2 and Figure 2c).

The emission was maintained and even slightly increased in ethanol during the investigation (Figure S5). However, transferring the sample to water causes the formation of aggregates and luminescence loss after a few days. (Figure S5). That occurred in the case of all the polymeric coatings investigated, even with the presence of amphiphilic block copolymers, where the hydrophobic block is bearing hydroxyl groups able to interact with the ZnO surface, with the hydrophilic block possibly providing colloidal stability in water. Therefore, these polymer coating agents are not able to prevent the final aggregation of the hybrids. For this reason, we investigated the use of silanes in combination with the hydroxylated polymers to improve colloidal stability and to preserve luminescence properties in water.

### 3.3. ZnO QDs with Silanes and a Combination of APTES Silane and Hydroxylated Polymers

**Scheme 2:**
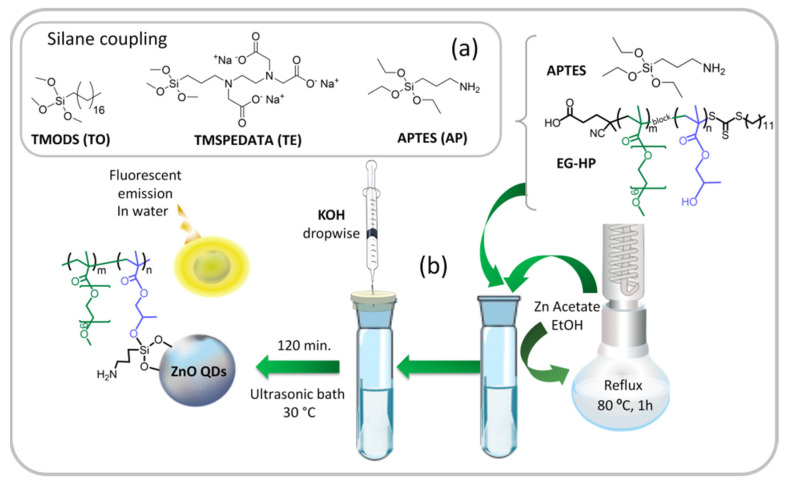
(**a**) Silane coupling agents for the synthesis of hybrid ZnO QDs. (**b**) Procedure for the synthesis of ZnO QDs protected with a mixture of APTES and EG-HP block copolymer.

**Figure 3:**
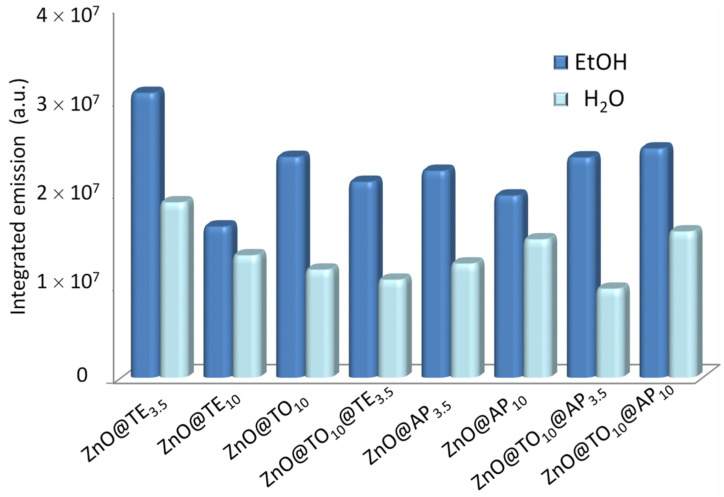
Integrated fluorescence emission as function of silane coupling, the combination of silanes, and their concentration.

The silanization of the ZnO QDs surface by means of alkoxysilanes [35,36] resulted in a robust method to preserve luminescence in an organic and aqueous medium. In this work, the three silanes displayed in Scheme 2 were explored; they exhibit different physico-chemical properties, so that TMODS (TO) is hydrophobic, APTES (AP) is hydrophilic, and TMSPEDATA (TE) is a salt and therefore very soluble in water. The reaction’s procedure is resumed in Scheme 2. Basically, silanes were added alone or in combination with hydroxylated polymers and block copolymers. The addition of KOH leads to silane hydrolysis and condensation, forming a dense cross-linked network around the ZnO nanoparticle, which will prevent the evolution of the ZnO QDs and a loss of luminescence over time. In addition, during condensation, silanol groups can react with hydroxyl groups of the polymers forming an organo-inorganic protective coating, as shown in Scheme 2. As a first step, ZnO QDs with one of the three silanes, or a combination of silanes shown in Scheme 2a, were prepared, and their photophysical properties are collected in Table 3. As it can be seen, λ_em.max._ lies between 550–561 nm (λ_exc._ = 365 nm) in ethanol, and the Φ_F_ is as high as 38% for APTES (ZnO@AP_3.5_). Increasing the silane concentration or the combination of them does not significantly increase the Φ_F_. In fact, it would appear that 3.5% of silane is enough to provide a good protection either in ethanol or in water (Figure 3).

Transferring the samples from ethanol to water, resulted in a slight decrease in the fluorescence emission compared to EtOH (Figure 3), but the solutions were still luminescent over time (Figure S6). Comparing the nanoparticles with the three silanes, ZnO@TE presented a low solubility in EtOH due to TMSPEDATA, while ZnO@TO nanoparticles presented low dispersion in water due to the high hydrophobicity of TMODS. For this reason and because they exhibited the highest Φ_F_, APTES in a 3.5% mol was chosen for its combination with hydroxylated polymers in the synthesis of hybrid ZnO QDs (Scheme 2b).

The results corresponding to the ZnO QDs synthesis in the presence of a combination of APTES (3.5% mol) and a selected polymer are shown in Table 4. The λ_em. max._ in ethanol and water present similar values as the ZnO QDs coated with polymers (Table 2) or silane (Table 3), indicating that the nature of the coating is not a determining factor, meaning that it is likely that other synthetic conditions, such as temperature or the KOH/Zn(CH_3_COO)_2_ ratio, have more influence over the λ_em. max_. The hydrodynamic sizes of these silane-polymer protected ZnO QDs in ethanol lie between 5–6 nm (Table 4), which is slightly smaller than the hydrodynamic sizes of ZnO QDs synthesized solely in the presence of the hydroxylated polymers (Table 2).

**Figure 4:**
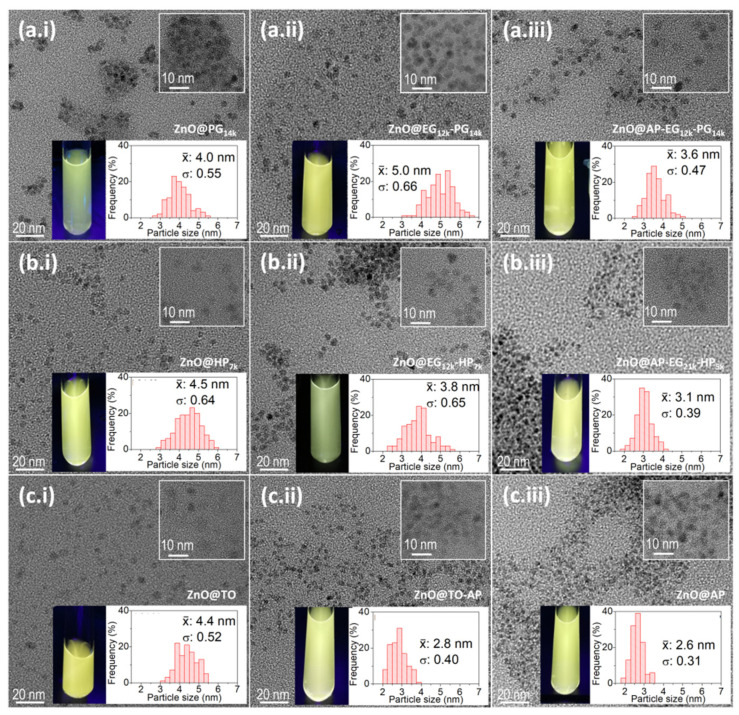
Representative TEM images of ZnO QDs with different coatings: (**a.i**) ZnO@PG_14k_; (**a.ii**) ZnO@EG_12k_-PG_14k_; (**a.iii**) ZnO@AP-EG_12k_-PG_14k_; (**b.i**) ZnO@HP_7k_; (**b.ii**) ZnO@EG_12k_-HP_7k_; (**b.iii**) ZnO@AP-EG_21k_-HP_5k_; (**c.i**) ZnO@TO; (**c.ii**) ZnO@TO-AP; and (**c.iii**) ZnO@AP. Inserts show photos under UV light (365 nm) and particle size histograms corresponding to the samples indicated in the figure.

TEM images and histograms of the particle distribution of representative samples with the different types of ligands are shown in Figure 4a–c, whereas in Figure S7, a chart comparing the mean size values of some representative samples is displayed. In Figure 4, it is observed in all cases that particles are spherical and well dispersed. The histograms displayed in Figure 4, determined by measuring the diameter of more than 120 ZnO nanoparticles, evidence that polymer-coated ZnO QDs exhibit broader distributions and larger sizes (3.8 to 5 nm, Figure 4 (a.i, a.ii, b.i, b.ii)) than silane-coated ZnO QDs (2.6 to 4.4 nm, Figure 4 (c.i, c.ii, c.iii)). The nanoparticles with hydrophobic TMODS silane present lager sizes than those synthesized with hydrophilic APTES or a combination of APTES and TMODS. In agreement with this result, the combination of APTES and hydroxylated copolymers resulted in ZnO QDs of smaller sizes than when they are coated solely with polymer (Figure 4a,b). The sizes estimated by TEM (Figure S7) matches with the results obtained from the XRD data (Figure S8), where a broadening of the diffraction peaks can be observed as the size of hybrid nanoparticles decreases. This behavior also indicates a reduction in the crystallinity of the samples with silane functionalization.

**Figure 5:**
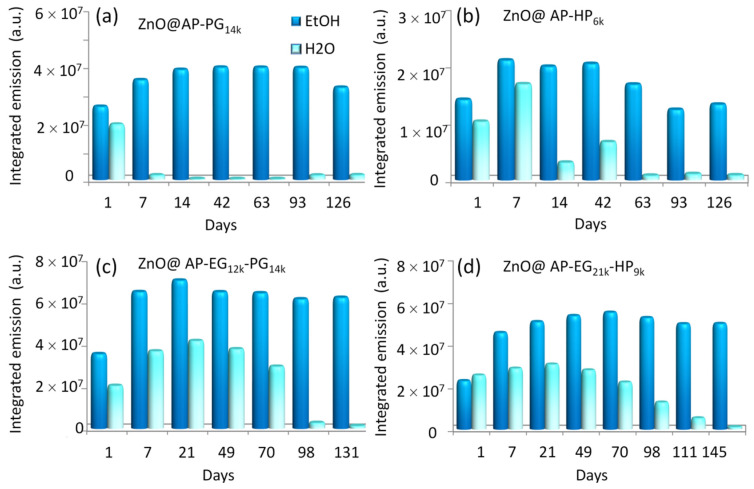
Integrated fluorescence emission as function of incubation time, for ZnO@AP-polymer in ethanol and water, synthesized with a combination of APTES (AP, 3.5% mol) and (**a**) PG_14k_ homopolymer, (**b**) HP_6k_ homopolymer, (**c**) EG_12k_-PG_14k_ block copolymer, and (**d**) EG_21k_-HP_9k_ block copolymer.

In Figure 5, the evolution of the integrated emission in ethanol and water are presented for selected ZnO@AP-polymers. For all samples investigated, it is observed that the emission noticeably increases in ethanol after 7 days, remaining practically unchanged during the entire period investigated. When transferring the hybrid ZnO QDs to water, the λ_em. max._ experiences a red shift compared to those in ethanol (Table 4), while the fluorescence emission decreases after 7 or 14 days in the case of the samples synthesized with APTES in combination with PG_14k_ or HP_6k_ homopolymers, respectively.

On the contrary, even more remarkable is the good protection offered by block copolymers in combination with APTES, as can be seen in Figure 5c,d, for ZnO@AP- EG_12k_-PG_14k_ and ZnO@AP-EG_21k_-HP_9k_, respectively. In Figure S9, images of these two ZnO QDs hybrids dispersed in water and ethanol evidences their colloidal stability in both media. As it can be seen in Figure 5c,d, the evolution of the fluorescence emission is similar in both media, ethanol and water, with it remaining stable for 70 days. After this time, the emissions in water are drastically extinguished. This result is promising when considering that in the absence of silane the emission was quenched after a few days (see Figure S5). Although these samples present high emission over time and are well dispersed in water, the increase in sample scattering when the sample was transferred to water was unavoidable. Since Φ_F_ is inversely proportional to absorbance (Equation (1)), this parameter drops in aqueous solution (i.e., for ZnO@AP-EG_21k_-HP_9k,_ Φ_F_ in H_2_O, it is 4.7%).

### 3.4. ZnO QDs as Sensors for Metal Detection

In previous works, it was shown that the loss of fluorescence experienced by ZnO QDs in the presence of certain metals can be used as sensors for these analytes in water [12,13,14,43]. In particular, a loss of fluorescence has been noticed in the presence of Cr^6+^, Cu^2+^ and Fe^3+^ [13], Fe^2+^ [12], and Cu^2+^ [14].

**Figure 6:**
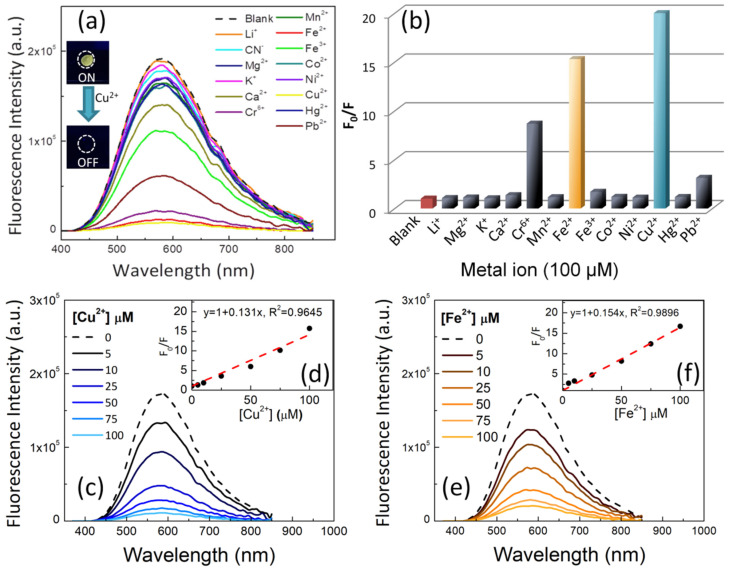
(**a**) Selectivity of ZnO@HP_6k_ towards different metal ions ([Metal ion] = 100 μM); (**b**) ratio between initial integrated emission and integrated emission (F_0_/F) of ZnO@HP_6k_ in the presence of 100 μM of the indicated metals; (**c**) decrease in fluorescence emission of ZnO@HP_6k_ as a function of Cu^2+^ concentration (5–100 μM); (**d**) Stern–Volmer plot corresponding to ZnO@HP_6k_ quenched by Cu^2+^; (**e**) decrease in fluorescence emission of ZnO@HP_6k_ as a function of Fe^2+^ concentration (5–100 μM); (**f**) Stern–Volmer plot corresponding to ZnO@HP_6k_ quenched by Fe^2+^.

Furthermore, certain hybrid ZnO QDs synthesized in this work were selected to explore to explore this environmental application. Specifically, the presence of HP_6k_ homopolymer (ZnO@HP_6k_) and ZnO coated with AP silane and EG_21k_-HP_9k_ block copolymer (ZnO@AP-EG_21k_-HP_9k_) were investigated as visible “turn-off” sensors for different metal ions. In Figure 6a,b, the decrease in fluorescence for ZnO@HP_6k_ as a function of metal type (100 μM aqueous solution) proves that nanohybrids are feasible for Fe^2+^, Cr^6+^, and Cu^2+^ detection compared to other metal ions tested (Li^+^, Mg^2+^, K^+^, Ca^2+^, Mn^2+^, Fe^3+^, Co^2+^, Ni^2+^, Hg^2+^, and Pb^2+^). Remarkably, a decrease in the fluorescence emission of almost 90% was detected in the presence of a 100 μM solution of Cu^2+^.

To verify the sensitivity of the ZnO QDs hybrids against quencher concentration, Stern–Volmer plots representing F_0_/F vs. concentration of Cu^2+^ and Fe^2+^ are displayed in Figure 6d,f, respectively. As shown in Figure 6, the fluorescence emission of the hybrid ZnO@HP_6k_ decreased significantly after their incubation, with increasing concentrations of Cu^2+^ and Fe^2+^ showing high sensitivity and a linear response in the range from 0 to 100 μM of the extinguisher metal. Similar results were obtained when the ZnO@AP-EG_21k_ -HP_9k_ nanohybrid was incubated in the presence of these metallic salts (Figure S10). It also reveals a noticeable selective fluorescence quenching against Cu^2+^, Fe^2+^, and Cr^6+^. It is interesting that both systems exhibit similar behavior despite the different polymeric composition and presence or absence of AP silane.

## 4. Conclusions

Fluorescent hybrid ZnO QDs with sizes between 4–5 nm were successfully synthesized using the sol-gel method and different polymers with hydroxyl groups as templates and ligands. By means of reversible-addition fragmentation chain transfer (RAFT) polymerization, multihydroxylated polymers and block copolymers with low dispersity were obtained to establish the influence of molecular weight, hydrophobic/hydrophilic balance, and polymer architecture on the colloidal and photophysical properties of the ZnO QD nanohybrids. A fluorescence enhancement occurred when ZnO QDs were synthesized in the presence of the hydroxylated polymers, especially when using block copolymers, although the nanoparticles aggregate when they are transferred to an aqueous solution. The stability of ZnO QDs in aqueous medium was achieved by means of a combination of organosilanes and hydroxylated polymers. In fact, these samples exhibited uniform and stable enhanced photoluminescence for nearly five months of investigation. The exceptional photoluminescent properties of these new ZnO QDs, coupled with their low price, means that they are expected to be used in biotechnological or environmental applications, such as metal detection. Indeed, the fluorescence quenching in the presence of some metals, such as for Fe^2+^, Cr^6+^, and Cu^2+^, makes the ZnO QDs promising materials for the detection of environmental contaminants.

## Figures and Tables

**Table 1:** Summary of reaction conditions for PG, HP homopolymers, EG macro-CTAs homopolymers, and EG-PG and EG-HP block copolymers synthesis; molecular weight parameters and conversion (%).

Homopolymer ^a^	[M]_0_/[CDTPA]	Time (h)	M_w_ ^b^ (g mol^−1^)	M_n_ ^b^ (g mol^−1^)	Ð ^b^	Conv. ^c^ (%)	M_n (theor.)_ ^d^ (g mol^−1^)
PG_7k_ ^e^	25	5	14,941	11,113	1.34	75	7435
PG_14k_	50	5	25,852	18,678	1.38	73	14,091
PG_17k_	75	5	28,156	20,451	1.38	58	16,716
HP_6k_	50	3	13,352	11,735	1.14	76	5882
HP_7k_	50	5	16,190	14,064	1.15	86	6603
HP_10k_	75	5	20,619	17,906	1.15	87	9811
EG_8k_	30	2	13,104	11,568	1.13	49	7754
EG_12k_	50	2	18,634	15,899	1.17	47	12,154
EG_21k_	50	4	25,068	21,494	1.17	81	20,654
**Block copolymer ^a^**	**[M]_0_/[EG]_0_**	**Time** **(h)**	**M_w_ ^b^** **(g mol^−1^)**	**M_n_ ^b^** **(g mol^−1^)**	**Ð ^b^**	**Conv. ^c^** **(%)**	**M_n (theor.)_ ^d^** **(g mol^−1^)**
EG_8k_-PG_8k_	50	3	19,591	16,451	1.19	42	15,629
EG_12k_-PG_14k_	50	3	39,189	30,383	1.29	77	26,591
EG_21k_-PG_14k_	50	3	43,736	33,850	1.29	72	33,904
EG_8k_-HP_5k_	50	3	21,543	17,715	1.22	75	13,160
EG_12k_-HP_6k_	50	3	27,621	22,635	1.22	82	18,065
EG_21k_-HP_5k_	50	3	32,373	27,407	1.18	71	25,522
EG_21k_-HP_9k_	75	5	37,167	32,021	1.16	80	29,054

^a^ PG = pPPGMA, HP = pHPMA, EG = pPEGMEMA. ^b^ M_w_, M_n_, and dispersity (Ð) determined by SEC using a series of PMMA standards. ^c^ Conversion (%) determined by ^1^H-NMR spectroscopy. ^d^ Molecular weight was calculated according to the equation: M_n (theor.)_ = ([Monomer]_0_/[CDTPA]_0_) × conversion × M_w_^Monomer^ + M_w_^CDTPA^, where M_w_^Monomer^ and M_w_^CDTPA^ are the Monomer and RAFT agent molecular weight, respectively. ^e^ Subscript indicates theoretical M_n_ in kg mol^−1^.

**Table 2:** Synthetic conditions, hydrodynamic diameter, and photophysical properties of hybrid ZnO QDs synthesized in the presence of multihydroxylated polymer ligands.

Sample	OH^-^/Zn^2+^ Ratio	Size ^a^ (nm)	λ_em. max._ (EtOH) (nm)	Φ_F_ (EtOH) (%)
ZnO@bare	-	837	554	1
ZnO@PG_7k_	1:1	8.0	556	16
ZnO@PG_14k_	1:1	7.6	549	30
ZnO@PG_17k_	1:1	5.9	547	22
ZnO@EG_8k_-PG_8k_	0.33:1	9.0	555	15
ZnO@EG_12k_-PG_14k_	0.33:1	6.7	557	23
ZnO@EG_21k_-PG_14k_	0.33:1	6.2	559	22
ZnO@HP_6k_	1:1	7.7	558	24
ZnO@HP_7k_	1:1	5.9	552	15
ZnO@HP_10k_	1:1	7.5	549	14
ZnO@EG_8k_-HP_5k_	0.35:1	5.1	563	36
ZnO@EG_12k_-HP_6k_	0.3:1	6.4	564	40
ZnO@EG_21k_-HP_5k_	0.2:1	6.7	557	43
ZnO@EG_21k_-HP_9k_	0.33:1	6.5	561	16

^a^ Hydrodynamic size values by number distribution in ethanol estimated by DLS measurements.

**Table 3:** Synthetic conditions, hydrodynamic diameter, and photophysical properties of ZnO QDs synthesized in the presence of three silane coupling agents: TMODS (TO), TMSPEDATA (TE), and APTES (AP).

ZnO@silane	Silane ^a^ (% mol)			Size ^b^ (nm)	λ_em. max._ (H_2_O) (nm)	λ_em. max._ (EtOH) (nm)	Φ_F_ (EtOH) (%)
	TE	TO	AP				
ZnO@TE_3.5_	3.5			9.4	557	551	29
ZnO@TE_10_	10			16.0	558	554	13
ZnO@TO_10_		10		7.0	571	550	30
ZnO@TO_10_@TE_3.5_	3.5	10		7.5	556	551	26
ZnO@ AP_3.5_			3.5	5.7	561	554	38
ZnO@ AP_10_			10	6.1	560	556	33
ZnO@ TO_10_@AP_3.5_		10	3.5	7.5	547	550	31
ZnO@ TO_10_@AP_10_		10	10	8.0	557	561	22

^a^ TE = TMSPEDATA; TO= TMODS; AP = APTES. ^b^ Hydrodynamic size values by number distribution in ethanol estimated by DLS measurements.

**Table 4:** Hydrodynamic diameter and photophysical properties of hybrid ZnO QDs synthesized in the presence of APTES (AP) and hydroxylated homopolymers and block copolymers.

Sample	OH^-^/Zn^2+^ ratio	Size ^a^ (nm)	λ_em. max_.(H_2_O) (nm)	λ_em. max._(EtOH) (nm)	Φ_F_ (EtOH) (%)
ZnO@AP-PG_14k_	1:1	4.7	564	549	28
ZnO@AP-EG_12k_-PG_14k_	0.33:1	5.9	567	551	41
ZnO@AP-HP_6k_	1:1	6.1	564	537	13
ZnO@AP-EG_21k_-HP_5k_	0.2:1	5.8	558	545	30
ZnO@AP-EG_21k_-HP_9k_	0.33:1	5.2	559	552	31

^a^ Hydrodynamic size values by number distribution in ethanol estimated by DLS measurements.

## Data Availability

Not applicable.

## Supplementary Materials

The following supporting information can be downloaded at: https://www.mdpi.com/article/10.3390/nano12193441/s1, Figure S1. ^1^H-NMR spectra corresponding to (a) hydrophilic pPEGMEMA (EG_21k_) used as macro-CTA for block copolymer synthesis (in CDCl_3_), (b) pHPMA homopolymer (HP_8k_) (in DMSO-d6), (c) pPPGMA homopolymer (PG_17k_) (in CDCl_3_), (d) ^1^H-^13^C HSQC spectrum of HP_6k_ in DMSO-d6, and (e) ^1^H-^13^C HSQC spectrum of PG_17k_ in CDCl_3_. Wrapped in dashed line the minor isomers of pHPMA and pPPGMA; Figure S2. SEC traces obtained for a series of EG-PG diblock copolymers (a) and EG-HP diblock copolymers (b) in DMF; Figure S3. ^1^H-NMR spectra corresponding to (a) pPEGMEMA-*b*-pHPMA (EG_21k_-HP_5k_) and (b) pPEGMEMA-*b*-pPPGMA (EG_21k_-PG_14k_) block copolymer, both in CDCl_3_. Wrapped in dashed line the minor isomers of pHPMA and pPPGMA; Figure S4. (a) Fluorescence quantum yield (Φ_F_) of ZnO QDs as a function of reaction temperature. (b) Evolution of the emitted florescence intensity as a function of reaction time for ZnO QDs synthesized at 30 °C; Figure S5. Time evolution of the integrated emission of the hybrid ZnO@polymer in ethanol and water; Figure S6. Time evolution of the integrated emission of water dispersions of ZnO QDs synthesized in the presence of TE, TO, and AP silanes or a combination of two of them; **Figure S7**. Variation in the ZnO QDs size determined by TEM as a function of the ligand or combination of ligands; Figure S8. X-ray diffraction patterns corresponding to representative ZnO QDs with polymer, silane, and a combination of silane-polymer coating. The size determined from XRD are included in the figure; Figure S9. (a) Absorbance (dashed lines) and emission fluorescence (λ_exc._ = 365 nm) (solid lines) bands from ethanol and aqueous solutions of ZnO@AP-EG_21k_-HP_9k._ (b) Images of ZnO@AP-EG_21k_-HP_9k_ and ZnO@AP-EG_12k_-PG_14k_ QDs in EtOH and water under UV (365 nm) and visible light; Figure S10. (a) Selectivity of ZnO@AP-EG_21k_-HP_9k_ towards different metal ions ([Metal ion] = 100 μM); (b) ratio between initial integrated emission and integrated emission (F_0_/F) of ZnO@AP-EG_21k_-HP_9k_ in the presence of 100 μM of the indicated metals; (c) Stern–Volmer plot corresponding to ZnO@AP-EG_21k_-HP_9k_ quenched by Cu^2+^; (d) decrease in fluorescence emission intensity of ZnO@AP-EG_21k_-HP_9k_ as a function of Cu^2+^ concentration (5–100 μM).
