# Supplementary material for: RAFT Hydroxylated Polymers as Templates and Ligands for the Synthesis of Fluorescent ZnO Quantum Dots
Source: Nanomaterials (Basel). 2022 Oct 1;12(19):3441. doi: 10.3390/nano12193441 (PMC9565916; doi:10.3390/nano12193441)
Supplement: Supplementary file 1 [file nanomaterials-12-03441-s001.zip › nanomaterials-1906236-supplementary.pdf]

nanomaterials

MDPI

Supplementary Materials for

RAFT hydroxylated polymers as templates and ligands for the synthesis of Fluorescent ZnO Quantum Dots

Leire San José, Olga García, Isabel Quijada-Garrido* and Mar López-González*

Group of Nanohybrids and Interactive Polymers. Instituto de Ciencia y Tecnología de Polímeros (ICTP-CSIC), C/ Juan de la Cierva, 3. 28006 Madrid, Spain

*Correspondence: iquijada@ictp.csic.es; mar@ictp.csic.es

Keyword: ZnO QDs; hydroxylated RAFT polymers; nanohybrids; fluorescent nanomaterials.

Figure S1. ^{1}H-NMR spectra corresponding to (a) hydrophilic pPEGMEMA (EG_{21k}) used as macro-CTA for block copolymer synthesis (in $CDCl_3$), (b) pHPMA homopolymer (HP_{8k}) (in DMSO-d6), (c) pPPGMA homopolymer (PG_{17k}) (in $CDCl_3$), (d) ^{1}H-^{13}C HSQC spectrum of HP_{6k} in DMSO-d6, and (e) ^{1}H-^{13}C HSQC spectrum of PG_{17k} in $CDCl_3$. Wrapped in dashed line the minor isomers of pHPMA and pPPGMA………………………………………………………………………….…. 2

Figure S2. SEC traces obtained for a series of EG-PG diblock copolymers (a) and EG-HP diblock copolymers (b) in DMF…………………………………………………………………...………... 3

Figure S3. ^{1}H-NMR spectra corresponding to (a) pPEGMEMA-*b*-pHPMA (EG_{21k}-HP_{5k}) and (b) pPEGMEMA-*b*-pPPGMA (EG_{21k}-PG_{14k}) block copolymer, both in $CDCl_3$. Wrapped in dashed line the minor isomers of pHPMA and pPPGMA…………………………………………………..…... 3

Figure S4. (a) Fluorescence quantum yield (Φ_F) of ZnO QDs as a function of reaction temperature. (b) Evolution of the emitted florescence intensity as a function of reaction time for ZnO QDs synthesized at 30 °C……………………………………………………………..……………………… 4

Figure S5. Time evolution of the integrated emission of the hybrid ZnO@polymer in ethanol and water…… 4

Figure S6. Time evolution of the integrated emission of water dispersions of ZnO QDs synthesized in the presence of TE, TO, and AP silanes or a combination of two of them as indicated……..……. 5

Figure S7. Variation in the ZnO QDs size determined by TEM as a function of the ligand or combination of ligands…………………………………………………………………………..……... 5

Figure S8. X-ray diffraction patterns corresponding to representative ZnO QDs with polymer, silane, and a combination of silane-polymer coating. The size determined from XRD are included in the figure….…………………………………………………………………………………………… 6

Figure S9. (a) Absorbance (dashed lines) and emission fluorescence ($\lambda_{exc.}$ = 365 nm) (solid lines) bands from ethanol and aqueous solutions of ZnO@AP-EG_{21k}-HP_{9k}. (b) Images of ZnO@AP-EG_{21k}-HP_{9k} and ZnO@AP-EG_{12k}-PG_{14k} QDs in EtOH and water under UV (365 nm) and visible light……………………………………………………………………………………………….. 6

Figure S10. (a) Selectivity of ZnO@AP-EG_{21k}-HP_{9k} towards different metal ions ([Metal ion]=100 μM); (b) ratio between initial integrated emission and integrated emission (F_0/F) of ZnO@AP-EG_{21k}-HP_{9k} in the presence of 100 μM of the indicated metals; (c) Stern–Volmer plot corresponding to ZnO@AP-EG_{21k}-HP_{9k} quenched by Cu^{2+}; (d) decrease in fluorescence emission intensity of ZnO@AP-EG_{21k}-HP_{9k} as a function of Cu^{2+} concentration (5–100 μM)………………………… 7

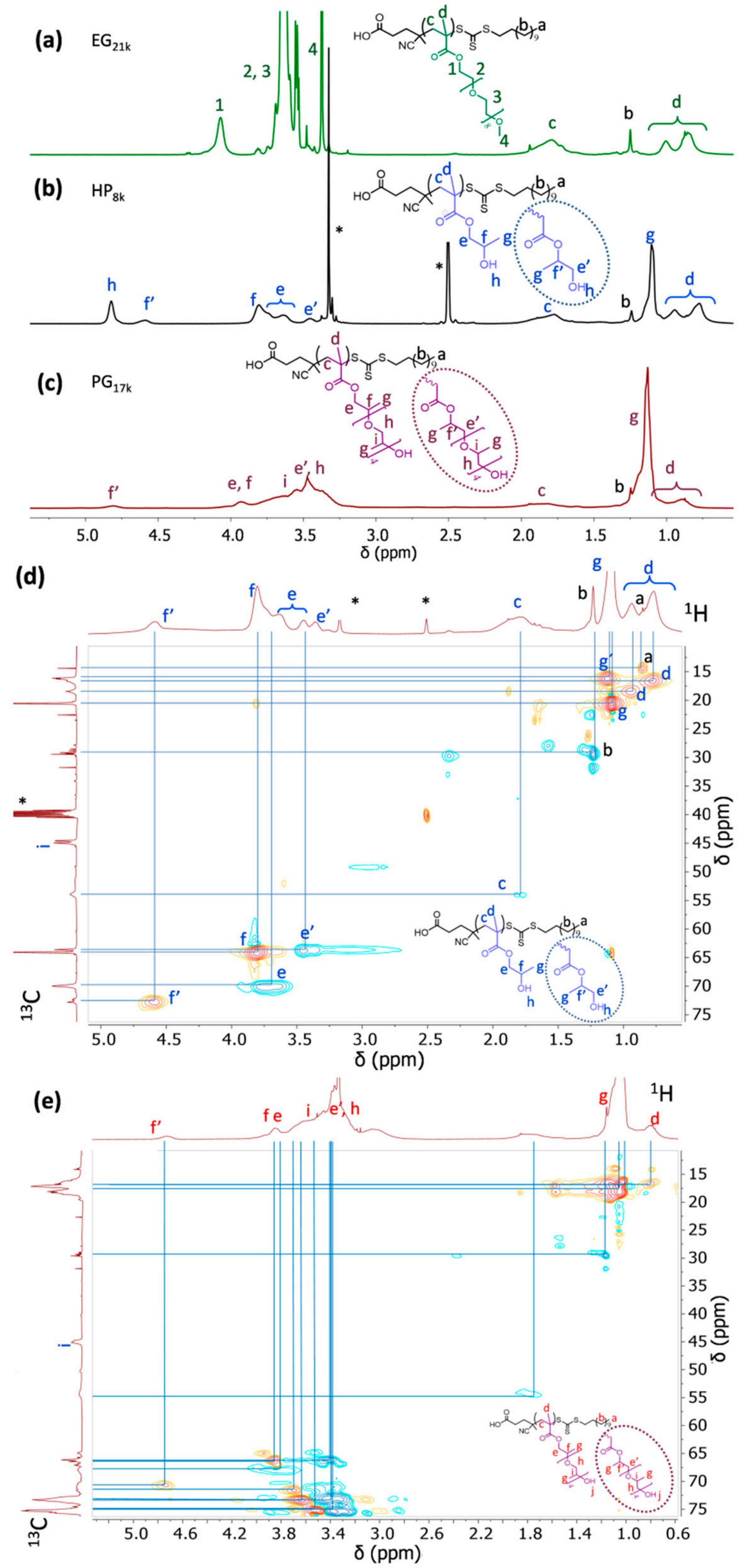

Figure S1. ^{1}H-NMR spectra corresponding to (a) hydrophilic pPEGMEMA (EG_{21k}) used as macro-CTA for block copolymer synthesis (in $CDCl_3$), (b) pHPMA homopolymer (HP_{8k}) (in DMSO-d6), (c) pPPGMA homopolymer (PG_{17k}) (in $CDCl_3$), (d) ^{1}H-^{13}C HSQC spectrum of HP_{6k} in DMSO-d6, and (e) ^{1}H-^{13}C HSQC spectrum of PG_{17k} in $CDCl_3$. Wrapped in dashed line the minor isomers of pHPMA and pPPGMA.

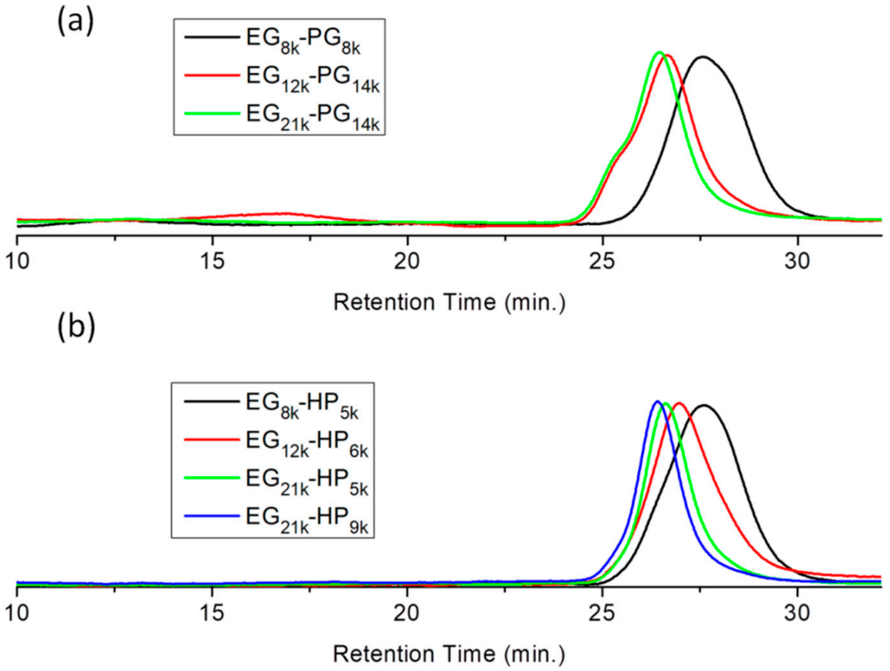

Figure S2. SEC traces obtained for a series of EG-PG diblock copolymers (a) and EG-HP diblock copolymers (b) in DMF.

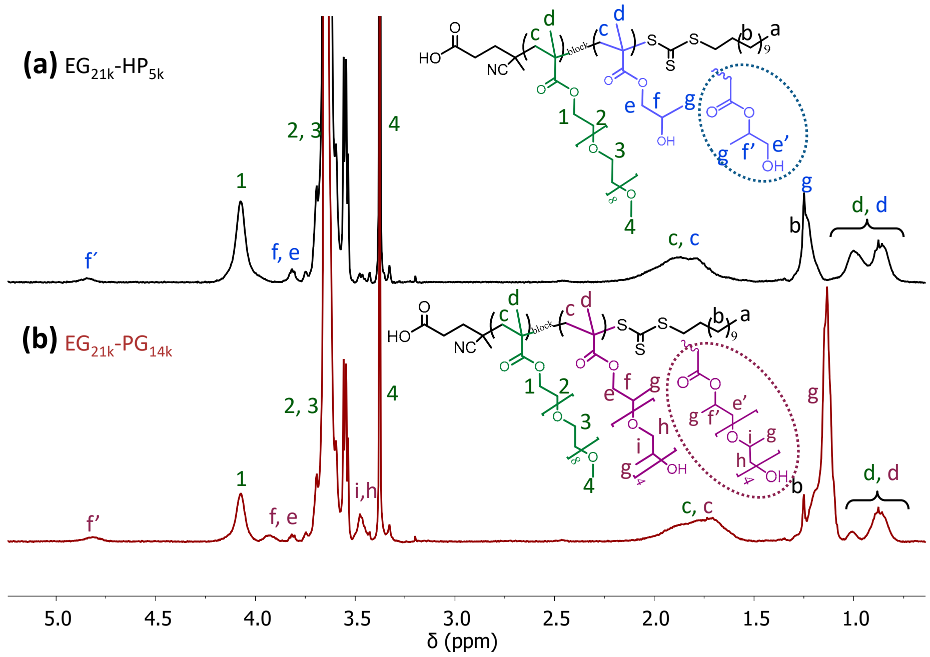

Figure S3. ^{1}H-NMR spectra corresponding to (a) pPEGMEMA-*b*-pHPMA (EG_{21k}-HP_{5k}) and (b) pPEGMEMA-*b*-pPPGMA (EG_{21k}-PG_{14k}) block copolymer, both in $CDCl_3$. Wrapped in dashed line the minor isomers of pHPMA and pPPGMA.

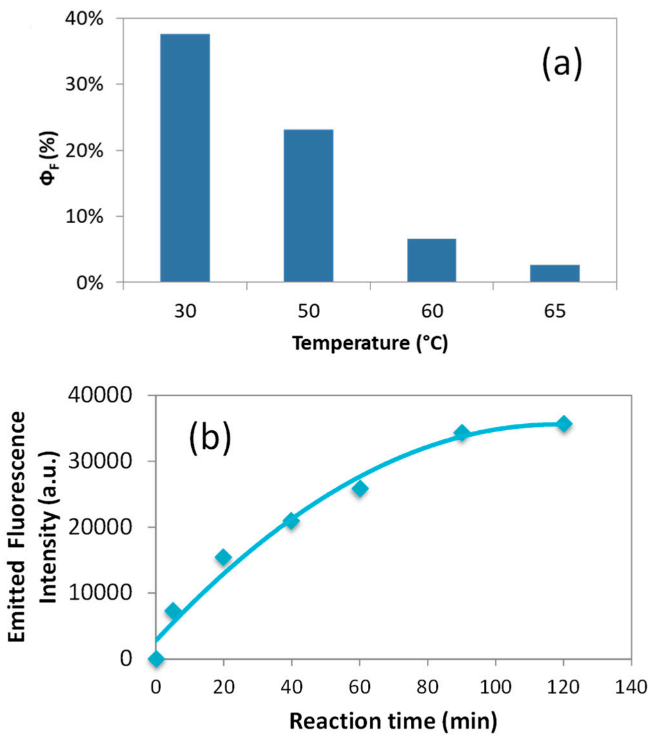

Figure S4. (a) Fluorescence quantum yield (Φ_F) of ZnO QDs as a function of reaction temperature. (b) Evolution of the emitted florescence intensity as a function of reaction time for ZnO QDs synthesized at 30 °C.

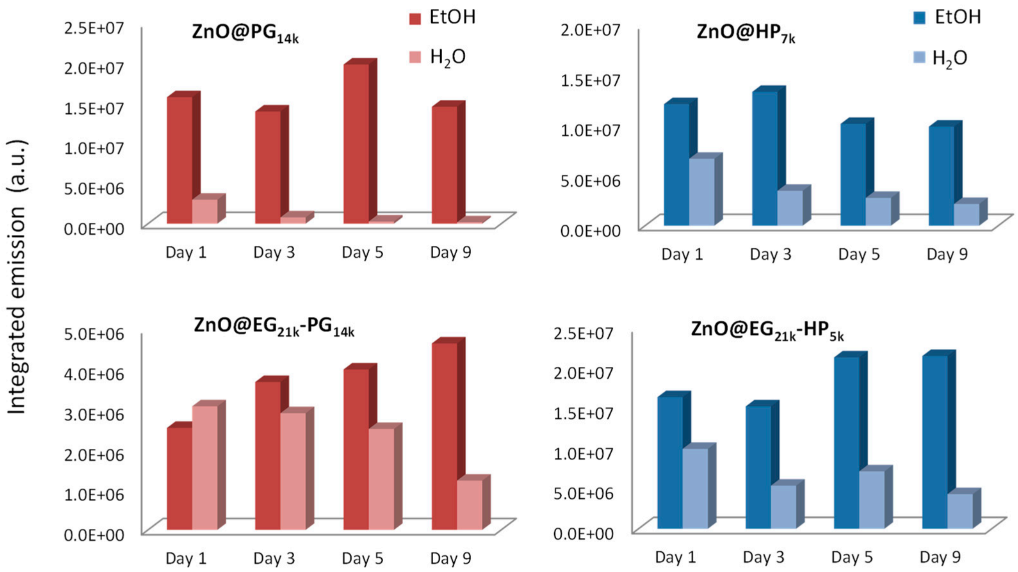

Figure S5. Time evolution of the integrated emission of the hybrid ZnO@polymer in ethanol and water.

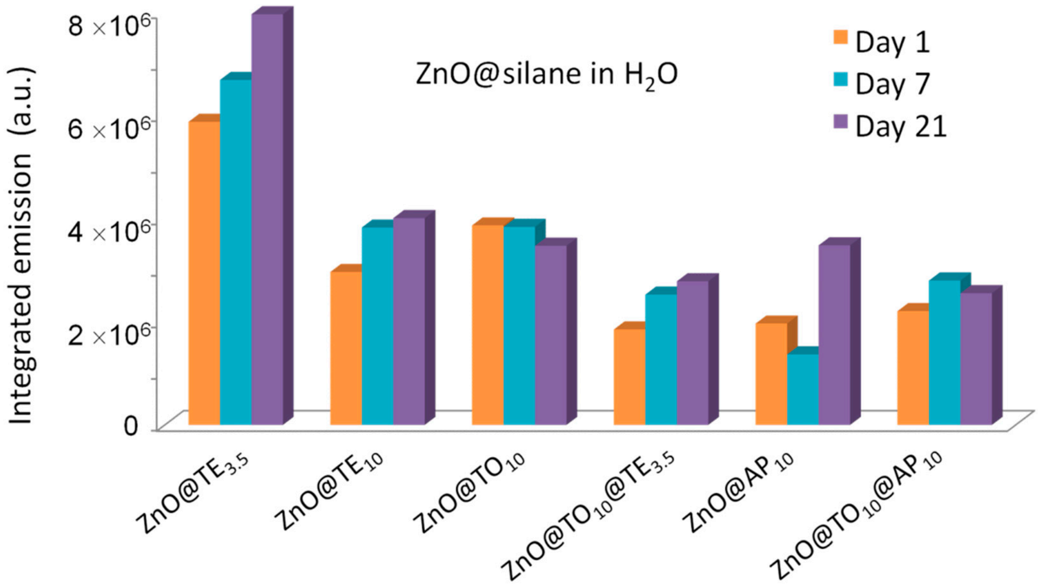

Figure S6. Time evolution of the integrated emission of water dispersions of ZnO QDs synthesized in the presence of TE, TO, and AP silanes or a combination of two of them as indicated.

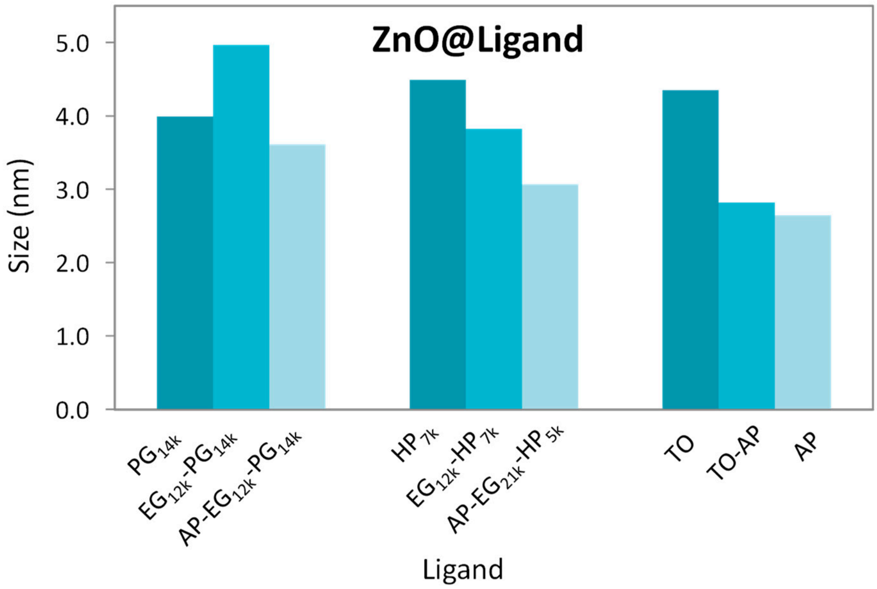

Figure S7. Variation in the ZnO QDs size determined by TEM as a function of the ligand or combination of ligands.

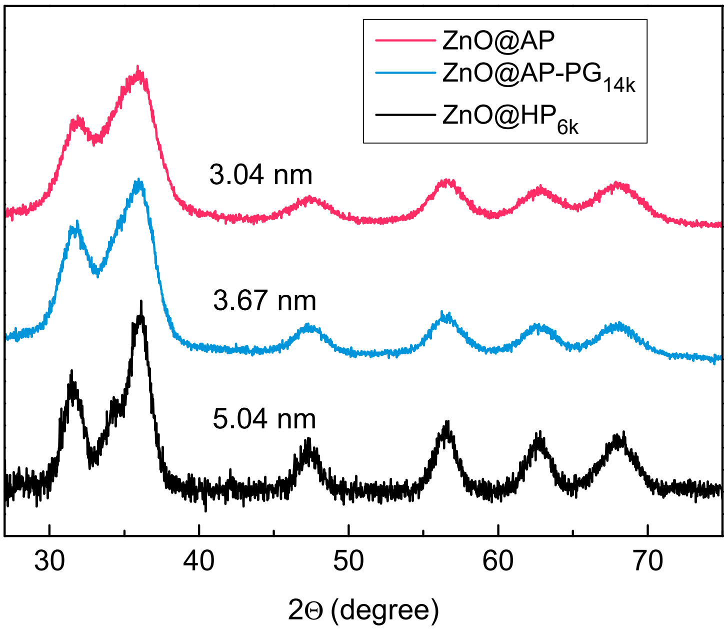

Figure S8. X-ray diffraction patterns corresponding to representative ZnO QDs with polymer, silane, and a combination of silane-polymer coating. The size determined from XRD are included in the figure.

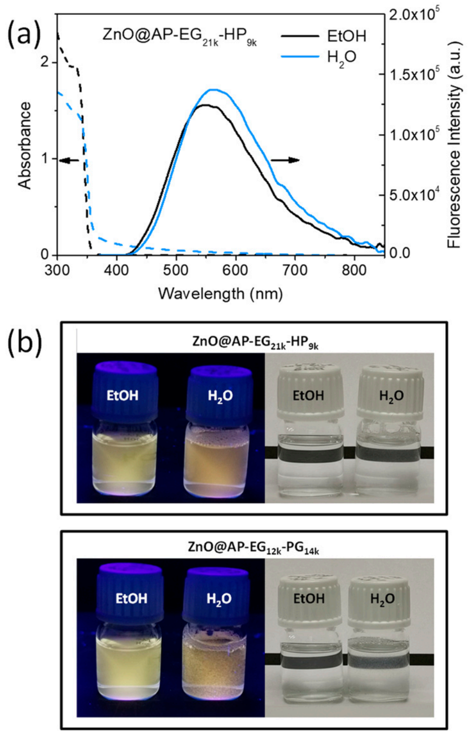

Figure S9. (a) Absorbance (dashed lines) and emission fluorescence ($\lambda_{exc.}$ = 365 nm) (solid lines) bands from ethanol and aqueous solutions of ZnO@AP-EG_{21k}-HP_{9k}. (b) Images of ZnO@AP-EG_{21k}-HP_{9k} and ZnO@AP-EG_{12k}-PG_{14k} QDs in EtOH and water under UV (365 nm) and visible light.

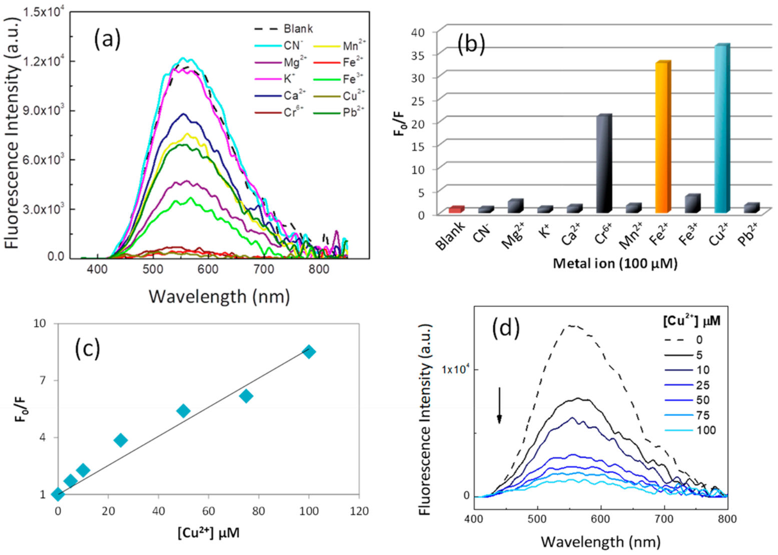

Figure S10. (a) Selectivity of ZnO@AP-EG_{21k}-HP_{9k} towards different metal ions ([Metal ion]=100 µM); (b) ratio between initial integrated emission and integrated emission (F_0/F) of ZnO@AP-EG_{21k}-HP_{9k} in the presence of 100 µM of the indicated metals; (c) Stern–Volmer plot corresponding to ZnO@AP-EG_{21k}-HP_{9k} quenched by Cu^{2+}; (d) decrease in fluorescence emission intensity of ZnO@AP-EG_{21k}-HP_{9k} as a function of Cu^{2+} concentration (5–100 µM).
